# Supplementary material for: Warming impacts potential germination of non-native plants on the Antarctic Peninsula
Source: Commun Biol. 2021 Mar 25;4:403. doi: 10.1038/s42003-021-01951-3 (PMC7994377; doi:10.1038/s42003-021-01951-3)
Supplement: Supplementary file 2 — Supplementary Information [file 42003_2021_1951_MOESM2_ESM.pdf]

Warming impacts potential germination of non-native plants on the Antarctic Peninsula

Stef Bokhorst, Peter Convey, Angélica Casanova-Katny and Rien Aerts

Supplementary Fig. 1 Season-specific diurnal microclimate patterns. Soil surface temperature and relative humidity at 10 cm height measured on Anchorage Island (Field) and the simulation of these meteorological conditions in climate chambers (2 °C and 7 °C) used in the germination experiment. Spring: mean November, Summer: mean of December-February, Autumn: mean of March. Please note that spring and autumn simulations only lasted one month while summer conditions were simulated for three months.

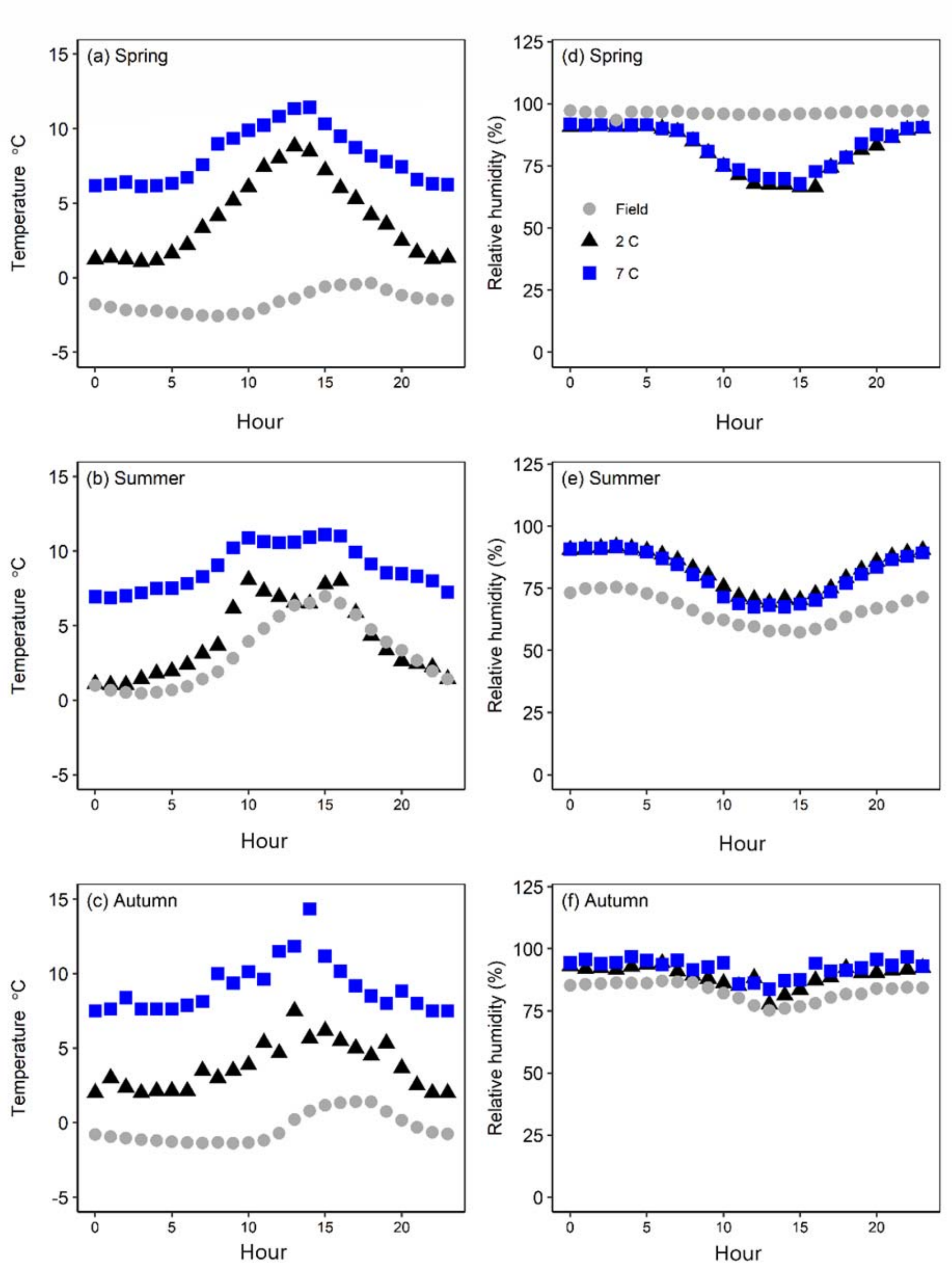

Supplementary Fig. 2 Monthly-specific diurnal patterns in photosynthetic active radiation. PAR values recorded on Anchorage Island (field) and the simulation of season-specific diurnal patterns (Spring: October-November, Summer: December-February, Autumn: March-April) in experimental chambers for germination studies. The high PAR values of November and December recorded in the field, could not be simulated with the experimental set-up and therefore, season specific diurnal patterns were used instead.

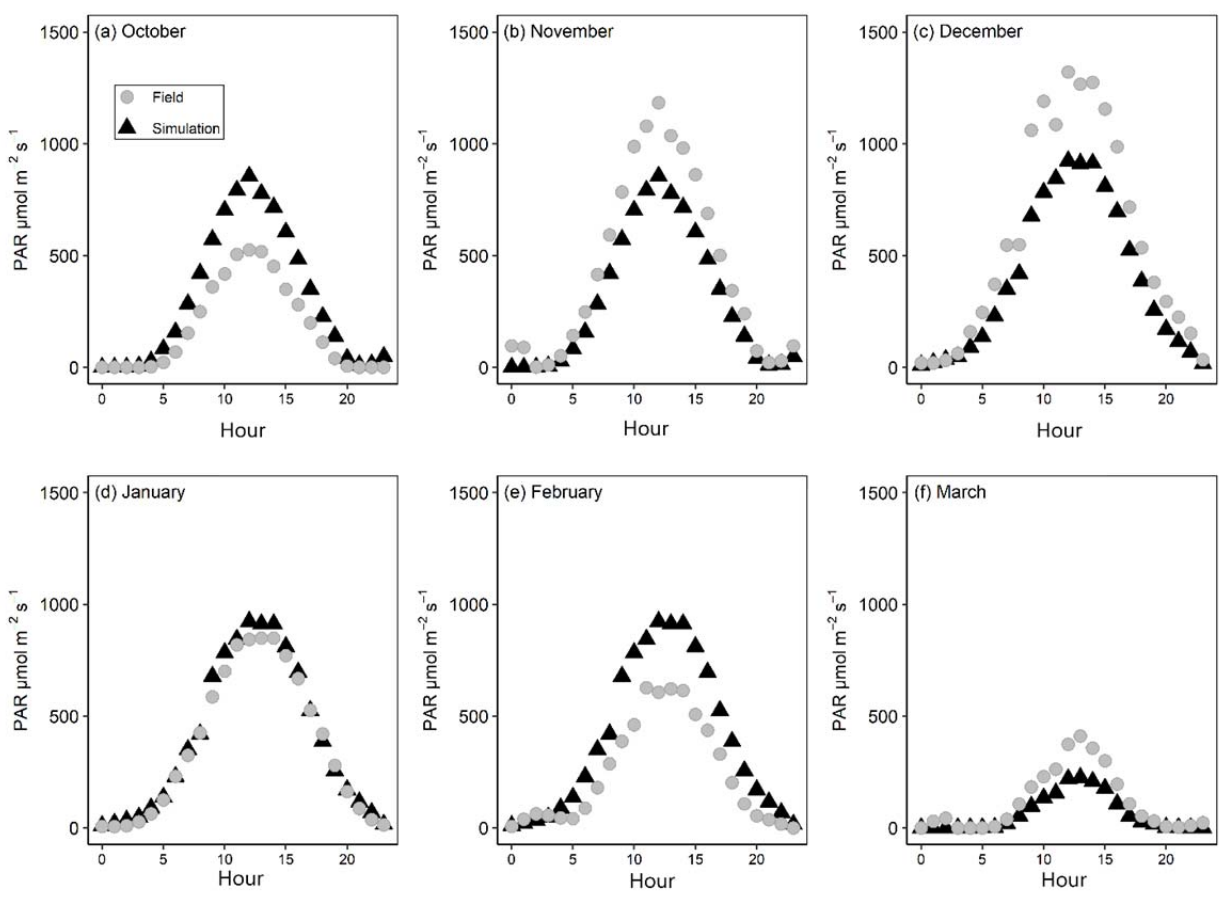

Supplementary Fig. 3 Annual degree day sums at sites along the Antarctic Peninsula. Correlation between latitude and soil surface degree day sums at sites along the Antarctic Peninsula. The equation was used to calculate degree day sums along the Antarctic Peninsula coastline.

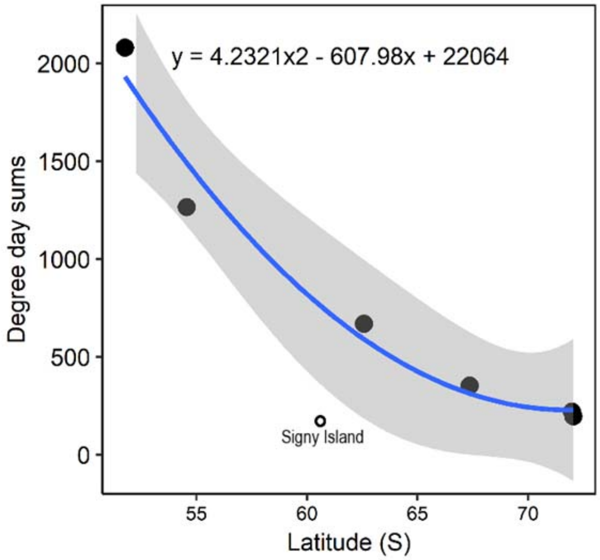

Supplementary Table 1 Plant species and number of seeds used per pot in the germination experiment. Seeds were sourced from Chile (1: Chileflora.com), The Netherlands (2 Exotischezaden.nl, 3: Cruydthoeck.nl, 4: collected from the field), UK (5: Plant-world-seeds.com) and Norway (6: Skogfrøverket.no). * = nitrogen fixing plant

Species	No. seeds/pot	Plant type	Family	source
*Astragalus cruckshanksii**	40	herb	Fabaceae	1
*Astragalus curvicaulis**	30	shrub	Fabaceae	1
Betula nana	25	shrub	Betulaceae	2
Blechnum penna marina	200	fern	Blechnaceae	1
Caiophora coronata	100	herb	Loasaceae	1
Calceolaria polyrhiza	30	herb	Scrophulariaceae	1
Cerastium arvense	50	herb	Caryophyllaceae	3
Draba polytricha	100	herb	Brassicaceae	5
Larix siberica	40	tree	Pinaceae	2
*Lupinus luteus**	3	herb	Fabaceae	3
Plantago lanceolata	100	herb	Plantaginaceae	3
Taraxacum officinale	50	herb	Asteraceae	4
*Trifolium repens**	100	herb	Leguminosaea	3
Dryas octopetala	60	shrub	Rosaceae	5
Empetrum rubrum	80	shrub	Empetraceae	1
Eucalyptus coccifera	25	tree	Myrtaceae	5
Eucalyptus perriniana	25	tree	Myrtaceae	2
Jasione montana	40	herb	Campanulaceae	5
Luzula spicata	100	rush	Juncaceaea	5
Luzula ulophylla	40	rush	Juncaceaea	5
Pinus sylvestris	20	tree	Pinaceae	6
Sedum album	40	succulent	Crassulaceae	3
Agrostis capillaris	100	grass	Poaceae	3
Deschampsia cespitosa	100	grass	Poaceae	3
Holcus lanatus	100	grass	Poaceae	3
Poa pratensis	100	grass	Poaceae	3

Supplementary Table 2 Species-specific degree day sum requirements for germination during the first and second simulated growing seasons. Plants were grown at Antarctic summer conditions (2 °C) or climate warming (7 °C) in Antarctic soil. Values are means of 1-5 replicate pots per treatment with SE between brackets. ‘-’ = absent. Significant differences between temperature are indicated by: * $p < 0.05$, ** $p < 0.01$, *** $p < 0.001$

		First growing season		Second growing season	
Plant type	species	2 °C	7 °C	2 °C	7 °C
grass	*Agrostis capillaris*	170 (30)	161 (25)	134 (25)	268 (34)**
grass	*Deschampsia cespitosa*	142 (19)	171 (25)	155 (18)	214 (0)*
grass	*Holcus lanatus*	92 (18)	81(14)	182 (5)	270 (55)
grass	*Poa pratensis*	194 (26)	166 (12)	173 (25)	220 (5)'
herb	*Caiophora coronata*	149 (12)	145 (12)	224 (43)	379 (115)
herb	*Calceolaria polyrhiza*	172 (25)	140 (15)	170 (17)	408 (180)
herb	*Cerastium arvense*	106 (11)	105 (12)	91 (0)	214 (0)***
herb	*Draba polytricha*	186 (29)	111 (12)*	78 (12)	183 (13)**
herb	*Jasione montana*	219 (25)	164 (6)*	147 (18)	353 (138)'
herb	*Plantago lanceolata*	253 (41)	147 (14)*	142 (32)	287 (30)*
herb	*Taraxacum officinale*	83 (13)	93 (20)	134 (25)	273 (42)*
rush	*Luzula spicata*	331 (45)	258 (29)	199 (22)	356 (40)*
rush	*Luzula ulophylla*	175 (29)	175 (49)	352 (0)	214 (-)***
N-fix	*Astragalus cruckshanksii*	299 (20)	106 (13)*	208 (59)	204 (10)
N-fix	*Lupinus luteus*	236 (100)	217 (30)	66 (-)	
N-fix	*Trifolium repens*	66 (6)	59 (9)	-	
succulent	*Sedum album*	202 (39)	140 (15)	117 (20)	220 (5)**
tree	*Pinus sylvestris*	-	621 (43)	-	-

Supplementary Table 3 Species-specific plant height (mm) at the end of two simulated growing seasons with an intervening simulated winter (-5 °C for 6 months) in Antarctic soil at Antarctic growing season temperatures (2 °C) and warming conditions (7 °C). Values are means of 1-5 replicate pots with SE between brackets. If SE are missing, seedling size was limited to one pot only. '-' = no plants emerged or survived until growth measurements were taken. Significant differences between temperature are indicated by: * $p < 0.05$, ** $p < 0.01$, *** $p < 0.001$, ' $p < 0.1$

		First growing season		Second growing season	
Plant type	Species	2 °C	7 °C	2 °C	7 °C
grass	*Agrostis capillaris*	27.6 (3.8)	65.8 (7.7)**	43.0 (14.6)	68.6 (8.0)
grass	*Deschampsia cespitosa*	32.8 (4.5)	71.4 (6.3)**	44.0 (6.8)	64.4 (8.2)'
grass	*Holcus lanatus*	34.6 (3.5)	69.4 (6.5)**	35.3 (7.8)	90.4 (18.0)*
grass	*Poa pratensis*	49.2 (2.1)	110.2	41.8 (15.2)	98.6 (8.6)*
herb	*Caiophora coronata*	7.2 (1.6)	12.2 (1.7)'	5.5 (0.5)	12.0 (3.6)
herb	*Calceolaria polyrhiza*	2.6 (0.2)	3.4 (0.4)	2.3 (0.3)	2.5 (0.5)
herb	*Cerastium arvense*	7.8 (1.9)	22.2 (3.2)**	14.5 (4.4)	25.6 (4.9)
herb	*Draba polytricha*	3.4 (0.7)	6.0 (1.6)	4.5 (0.5)	3.8 (0.8)
herb	*Jasione montana*	2.0 (0.0)	4.8 (1.1)*	2.8 (0.8)	3.0 (0.7)
herb	*Plantago lanceolata*	14.6 (2.7)	58.0 (3.9)***	14.7 (0.9)	41.2 (11.3)*
herb	*Taraxacum officinale*	9.0 (2.3)	26.6 (5.5)'	13.0 (4.6)	12.3 (5.3)
rush	*Luzula spicata*	4.5 (1.5)	16.2 (3.2)*	4.5 (0.5)	24.3 (5.7)*
rush	*Luzula ulophylla*	4.0 (1.2)	7.2 (1.3)	3.5 (1.5)	8 (-)
shrub	*Astragalus curvicaulis*	9.0 (-)	-	-	27.5 917.5)
N-fix	*Astragalus*	12.5 (7.5)	26.0 (14.0)	12.7 (1.3)	26.4 (2.9)*
N-fix	*Lupinus luteus*	3.0 (-)	40.3 (24.4)'	-	-
N-fix	*Trifolium repens*	8.8 (2.0)	13.8 (2.5)	-	-
succulent	*Sedum album*	3.0 (0.5)	9.2 (1.0)**	3.8 (0.7)	6.6 (0.9)*
tree	*Pinus sylvestris*	-	10 (2.7)	-	-

Supplementary Table 4 Species-specific number of shoots/leaves grown from seeds during two simulated growing seasons with an intervening simulated winter (-5 °C for 6 months) in Antarctic soil at Antarctic growing season temperatures (2 °C) and warming conditions (7 °C). Values are means of 1-5 replicate pots with SE between brackets. If SE are missing, seedling emergence was limited to one pot only. '-' = no plants emerged or survived until growth measurements were taken. Leaves were counted for: grass, herb, rushes and N-fixing plants while for shrubs, trees and *Sedum album* we counted shoots. Significant differences between temperature are indicated by: * $p < 0.05$, ** $p < 0.01$, *** $p < 0.001$

		First growing season		Second growing season	
Plant type	Species	2 °C	7 °C	2 °C	7 °C
grass	*Agrostis capillaris*	82.8 (13.9)	62.8 (13.9)	24.3 (5.9)	17.6 (5.5)
grass	*Deschampsia cespitosa*	29.0 (4.0)	22.0 (2.7)	4.5 (2.0)	21.2 (5.5)*
grass	*Holcus lanatus*	15.8 (2.8)	19.6 (1.1)	5.7 (1.2)	11.2 (3.2)
grass	*Poa pratensis*	28.4 (6.6)	20.2 (4.0)	15.5 (4.3)	26.2 (5.8)
herb	*Caiophora coronata*	69.4 (5.9)	35.4 (6.5)**	4.8 (1.7)	2.4 (0.4)
herb	*Calceolaria polyrhiza*	32.6 (10.3)	17.0 (2.9)	8.0 (2.3)	5.0 (2.0)
herb	*Cerastium arvense*	15.2 (3.7)	18.6 (1.0)	4.3 (0.9)	9.0 (2.2)
herb	*Draba polytricha*	2.0 (0.6)	2.3 (0.7)	2.0 (1.0)	1.5 (0.3)
herb	*Jasione montana*	35.3 (9.6)	43.4 (9.2)	9.5 (3.1)	7.5 (3.4)
herb	*Plantago lanceolata*	6.8 (1.7)	17.0 (1.7)*	9.3 (5.9)	5.2 (0.4)
herb	*Taraxacum officinale*	18.8 (5.4)	18.2 (2.9)	3.0 (1.5)	3.5 (1.2)
rush	*Luzula spicata*	3.3 (1.0)	10.2 (2.0)*	7.0 (1.0)	5.3 (1.3)
rush	*Luzula ulophylla*	8.7 (2.7)	18.6 (4.8)	1.5 (0.5)	3 (-)
shrub	*Astragalus curvicaulis*	1 (-)	-	-	1.0 (0.0)
N-fix	*Astragalus cruckshanksii*	1.5 (0.5)	2.0 (1.0)	2.3 (0.7)	3.0 (0.8)
N-fix	*Lupinus luteus*	1 (-)	1.7 (0.7)	-	-
N-fix	*Trifolium repens*	12.2 (5.4)	18.2 (2.9)*	-	-
succulent	*Sedum album*	13.2 (3.6)	27.4 (8.8)	12.4 (5.1)	8.0 (1.2)
tree	*Pinus sylvestris*	-	1.8 (0.3)	-	-

Supplementary Table 5 Seasonal soil surface temperature, degree days sums and calculated degree day sums under warming scenario (+3°C and +5 °C) at various sites along the Antarctic Peninsula, and southern Chile and the Falkland Islands.

			Years data	Temperature (°C)		Degree day sums		
			available	Winter	Summer	Ambient	(+3°C)	(+5°C)
Site name	Latitude (S)	Longitude (W)	(range)	Jun-Aug	Dec-Feb	Oct-March	Oct-March	Oct-March
Coal Nunatak	72.05	68.55	2008-2018	-14.5	0.8	196	340	520
Mars Oasis	71.98	68.38	2001-2019	-16.9	2.3	220	503	779
Anchorage Island	67.36	62.21	2001-2007	-7.9	3.9	353	761	1068
Deception Island	62.58	60.39	2019-2020	-3.3	4.9	670	1093	1437
Signy Island	60.72	45.60	2004-2005	-7.0	1.9	252	746	1112
Cerro Bandera	54.56	67.37	2019	-2.2	8.1	1266	1810	2174
Falkland Islands	51.76	59.03	2004-2005	2.3	12.0	2081	2224	2567

Supplementary Table 6 Species-specific degree day sum requirements for germination during the first and second simulated growing seasons. Plants were grown at Antarctic summer conditions (2 °C) or climate warming (7 °C) in potting soil. Values are means of 1-5 replicate pots per treatment with SE between brackets. '-' = absent. Significant differences between temperature are indicated by: * $p < 0.05$, ** $p < 0.01$, *** $p < 0.001$

		First growing season		Second growing season	
Plant type	Species	2 °C	7 °C	2 °C	7 °C
grass	*Agrostis capillaris*	169 (12)	182 (20)	240 (76)	324 (97)
grass	*Deschampsia cespitosa*	163 (16)	174 (17)	250 (75)	228 (0)
grass	*Holcus lanatus*	119 (10)	62 (9)	382 (0)	228 (0)
grass	*Poa pratensis*	188 (15)	209 (26)	222 (23)	228 (0)
herb	*Caiophora coronata*	161 (7)	128 (10)	148 (15)	301 (60)
herb	*Calceolaria polyrhiza*	206 (18)	228 (44)	126 (8)	228 (0)
herb	*Cerastium arvense*	101 (14)	130 (14)	290 (92)	228 (0)
herb	*Draba polytricha*	113 (25)	129 (22)	102 (18)	200 (16)
herb	*Jasione montana*	163 (15)	169 (6)	-	255
herb	*Plantago lanceolata*	177 (20)	161 (23)	210 (12)	301 (54)
herb	*Taraxacum officinale*	116 (7)	80 (11)	162 (53)	343 (115)
rush	*Luzula spicata*	258 (53)	255 (33)	256 (73)	433 (12)
rush	*Luzula ulophylla*	191 (17)	202 (52)	-	-
N-fix	*Astragalus cruckshanksii*	199 (107)	331 (90)	118 (26)	200 (17)
N-fix	*Lupinus luteus*	148 (21)	237 (18)	-	-
N-fix	*Trifolium repens*	117 (29)	84 (22)	-	421
succulent	*Sedum album*	148 (5)	134 (7)	191 (55)	246 (74)
shrub	*Dryas octopetala*	211 (-)	191 (-)	-	-
tree	*Eucalyptus perriniana*	-	-	-	-
tree	*Pinus sylvestris*	-	532 (102)	-	-

Supplementary Table 7 Species-specific plant height (mm) at the end of two simulated growing seasons with an intervening simulated winter (-5 °C for 6 months) in potting soil at Antarctic growing season temperatures (2 °C), warming conditions (7 °C) and a seedling control of 15 °C. Values are means of 1-5 replicate pots with SE between brackets. If SE are missing, seedling size was limited to one pot only. '-' = no plants emerged or survived until growth measurements were taken.

		First growing season			Second growing season	
Plant type	Species	2 °C	7 °C	15 °C	2 °C	7 °C
grass	*Agrostis capillaris*	55.6 (10.2)	93.4 (5.9)	92.8 (7.3)	23.5 (11.5)	30.0 (20.0)
grass	*Deschampsia cespitosa*	49.8 (8.7)	103.8 (13.6)	141.0 (14.1)	37.7 (16.2)	54.2 (6.9)
grass	*Holcus lanatus*	60.4 (3.7)	123.4 (7.5)	176.0 (14.6)	17.5 (7.5)	43.8 (8.2)
grass	*Poa pratensis*	60.4 (4.6)	138.4 (11.6)	238.0 (17.2)	34.8 (2.1)	98.6 (18.0)
herb	*Astragalus curvicaulis*	-	-	61.7 (20.9)	10.0 (-)	12.0 (-)
herb	*Caiophora coronata*	7.8 (1.4)	18.0 (3.9)	21.0 (3.3)	4.6 (1.0)	7.8 (1.7)
herb	*Calceolaria polyrhiza*	4.8 (1.4)	5.4 (1.4)	73.5 (33.3)	2.8 (0.2)	5.0 (-)
herb	*Cerastium arvense*	9.4 (2.9)	44.6 (4.3)	75.6 (11.9)	7.0 (2.0)	45.6 (7.6)
herb	*Draba polytricha*	6.5 (1.5)	7.5 (2.5)	9.3 (0.5)	5 (-)	9.4 (2.2)
herb	*Jasione montana*	2.8 (0.6)	13.0 (2.3)	97.0 (9.3)	-	3.5 (0.5)
herb	*Plantago lanceolata*	29.2 (2.7)	71.8 (2.6)	36.0 (4.3)	14.5 (5.4)	34.5 (2.1)
herb	*Taraxacum officinale*	20.6 (2.1)	35.2 (2.4)	16.8 (2.2)	5 (-)	13.3 (3.3)
N-fix	*Astragalus cruckshanksii*	10.0 (-)	22.8 (7.1)	92.8 (15.7)	15.0 (2.2)	38.0 (6.0)
N-fix	*Lupinus luteus*	13.0 (-)	46.7 (20.5)	195.0 (13.4)	-	-
N-fix	*Trifolium repens*	15.2 (2.6)	32.4 (4.6)	59.0 (13.1)	-	-
rush	*Luzula spicata*	7.3 (0.3)	39.4 (7.2)	62.0 (7.6)	7.8 (0.5)	27.5 (7.5)
rush	*Luzula ulophylla*	5.7 (1.8)	12.8 (2.7)	25.4 (1.4)	-	-
shrub	*Dryas octopetala*	5.0 (-)	35.0 (-)	4 (-)	-	-
succulent	*Sedum album*	3.8 (0.7)	17.6 (3.8)	29 (4.3)	4.0 (0.7)	4.0 (2.0)
tree	*Eucalyptus coccifera*	-	-	35.0 (-)	-	-
tree	*Eucalyptus perriniana*	-	-	24.0 (12.9)	-	-
tree	*Pinus sylvestris*	-	16.0 (6.0)	20.0 (-)	-	-

Supplementary Table 8 Species-specific number of shoots/leaves grown from seeds during two simulated growing seasons with an intervening simulated winter (-5 °C for 6 months) in potting soil at Antarctic growing season temperatures (2 °C) and warming conditions (7 °C). Values are means of 1-5 replicate pots with SE between brackets. If SE are missing, seedling emergence was limited to one pot only. '-' = no plants emerged or survived until growth measurements were taken. Leaves were counted for: grass, herb, rushes and N-fixing plants while for shrubs, trees and *Sedum album* we counted shoots.

		First growing season		Second growing season	
Plant type	species	2 °C	7 °C	2 °C	7 °C
grass	*Agrostis capillaris*	81.0 (9.3)	63.6 (4.4)	9.5 (5.5)	23.5 (21.5)
grass	*Deschampsia cespitosa*	24.0 (2.9)	27.8 (1.2)	3.3 (0.7)	29.0 (4.6)
grass	*Holcus lanatus*	21.2 (3.2)	23.2 (2.4)	3.5 (2.5)	7.8 (1.3)
grass	*Poa pratensis*	19.6 (4.8)	25.5 (5.0)	27.3 (15.0)	12.2 (1.9)
herb	*Astragalus curvicaulis*	-	-	1 (-)	1 (-)
herb	*Caiophora coronata*	50.8 (7.5)	27.6 (9.0)	4.8 (2.2)	2.8 (0.8)
herb	*Calceolaria polyrhiza*	23.2 (5.0)	12.6 (3.4)	16.2 (5.2)	1 (-)
herb	*Cerastium arvense*	16.8 (3.0)	11.8 (1.2)	1.5 (0.5)	10.0 (1.8)
herb	*Draba polytricha*	1 (-)	5.0 (3.0)	1 (-)	1.8 (0.4)
herb	*Jasione montana*	38.4 (6.8)	27.0 (7.1)	-	1.5 (0.5)
herb	*Plantago lanceolata*	21.2 (2.7)	19.8 (3.1)	7.3 (2.1)	4.3 (1.1)
herb	*Taraxacum officinale*	24.6 (4.4)	21.4 (2.1)	3.4 (0.7)	3.6 (0.5)
rush	*Luzula spicata*	15.7 (3.3)	16.6 (3.3)	7.5 (4.2)	7.0 (1.1)
rush	*Luzula ulophylla*	5.7 (1.7)	9.5 (1.5)	-	-
N-fix	*Astragalus cruckshanksii*	1 (-)	3.0 (1.0)	1 (-)	1.0 (0.0)
N-fix	*Lupinus luteus*	1 (-)	1.7 (0.3)	-	-
N-fix	*Trifolium repens*	13.8 (2.5)	20.2 (1.8)	-	-
shrub	*Dryas octopetala*	1 (-)	1 (-)	-	-
succulent	*Sedum album*	28.6 (7.5)	22.6 (5.1)	10.3 (4.8)	2.5 (0.5)
tree	*Eucalyptus coccifera*	-	-	-	-
tree	*Eucalyptus perriniana*	-	-	-	-
tree	*Pinus sylvestris*	-	1.0 (0.0)	-	-
