# Supplementary material for: Warming impacts potential germination of non-native plants on the Antarctic Peninsula
Source: Commun Biol. 2021 Mar 25;4:403. doi: 10.1038/s42003-021-01951-3 (PMC7994377; doi:10.1038/s42003-021-01951-3)
Supplement: Supplementary file 3 — Reporting Summary [file 42003_2021_1951_MOESM3_ESM.pdf]

Corresponding author(s): Stef Bokhorst
Last updated by author(s): Dec 7, 2020

Reporting Summary

Nature Research wishes to improve the reproducibility of the work that we publish. This form provides structure for consistency and transparency in reporting. For further information on Nature Research policies, see our Editorial Policies and the Editorial Policy Checklist.

Statistics

For all statistical analyses, confirm that the following items are present in the figure legend, table legend, main text, or Methods section.

n/a	Confirmed	
[ ]	[x]	The exact sample size (*n*) for each experimental group/condition, given as a discrete number and unit of measurement
[ ]	[x]	A statement on whether measurements were taken from distinct samples or whether the same sample was measured repeatedly
[ ]	[x]	The statistical test(s) used AND whether they are one- or two-sided<br>*Only common tests should be described solely by name; describe more complex techniques in the Methods section.*
[x]	[ ]	A description of all covariates tested
[ ]	[x]	A description of any assumptions or corrections, such as tests of normality and adjustment for multiple comparisons
[ ]	[x]	A full description of the statistical parameters including central tendency (e.g. means) or other basic estimates (e.g. regression coefficient) AND variation (e.g. standard deviation) or associated estimates of uncertainty (e.g. confidence intervals)
[ ]	[x]	For null hypothesis testing, the test statistic (e.g. *F*, *t*, *r*) with confidence intervals, effect sizes, degrees of freedom and *P* value noted<br>*Give P values as exact values whenever suitable.*
[x]	[ ]	For Bayesian analysis, information on the choice of priors and Markov chain Monte Carlo settings
[x]	[ ]	For hierarchical and complex designs, identification of the appropriate level for tests and full reporting of outcomes
[x]	[ ]	Estimates of effect sizes (e.g. Cohen's *d*, Pearson's *r*), indicating how they were calculated

Our web collection on statistics for biologists contains articles on many of the points above.

Software and code

Policy information about availability of computer code

Data collection	no software was used
Data analysis	R version 4.0.2 (2020-06-22) -- "Taking Off Again"<br>Copyright (C) 2020 The R Foundation for Statistical Computing

For manuscripts utilizing custom algorithms or software that are central to the research but not yet described in published literature, software must be made available to editors and reviewers. We strongly encourage code deposition in a community repository (e.g. GitHub). See the Nature Research guidelines for submitting code & software for further information.

Data

Policy information about availability of data

All manuscripts must include a data availability statement. This statement should provide the following information, where applicable:

- Accession codes, unique identifiers, or web links for publicly available datasets
- A list of figures that have associated raw data
- A description of any restrictions on data availability

Publicly available datasets of micro climate are referenced in the literature list. All other data generated or analysed during this study are included in this published article (and its supplementary files) and has been deposited at: Dataset - Plant germination in Antarctica | Netherlands Polar Data Center (npdc.nl)

Field-specific reporting

Please select the one below that is the best fit for your research. If you are not sure, read the appropriate sections before making your selection.

- [ ] Life sciences
- [ ] Behavioural & social sciences
- [x] Ecological, evolutionary & environmental sciences

For a reference copy of the document with all sections, see nature.com/documents/nr-reporting-summary-flat.pdf

Ecological, evolutionary & environmental sciences study design

All studies must disclose on these points even when the disclosure is negative.

Study description

Quantification of germination and plant growth of 26 non-native vascular plants in Antarctic soil at realistic simulated micro climate conditions and a warming scenario under laboratory settings

Research sample

To test whether seeds from non-native species could germinate and grow in Antarctic soil under current conditions we conducted a climate chamber experiment where germination time and subsequent plant growth of 26 species was quantified.

Species selections were based on either the proximity of native distributions to Antarctica (i.e. southern South America, southern Australia and New Zealand), or on possession of ruderal characteristics and boreal/Arctic/Alpine provenance and included the following species:

Astragalus cruckshanksii*
Astragalus curvicaulis*
Betula nana
Blechnum penna marina
Caiophora coronata
Calceolaria polyrhiza
Cerastium arvense
Draba polytricha
Larix siberica
Lupinus luteus*
Plantago lanceolata
Taraxacum officinale
Trifolium repens*
Dryas octopetala
Empetrum rubrum
Eucalyptus coccifera
Eucalyptus perriniana
Jasione montana
Luzula spicata
Luzula ulophylla
Pinus sylvestris
Sedum album
Agrostis capillaris
Deschampsia cespitosa
Holcus lanatus
Poa pratensis

Sampling strategy

We used 5 replicate pots for each species and temperature treatment to quantify germination speed and plant growth. Replication no. was primarily determined by available space in climate chambers but sufficient to accurately quantify the required parameters.

Data collection

From the onset of spring, when lights came on, we noted the number of days required for the first seedling to germinate in each experimental pot at 3-4 day intervals until the end of the first growing season. From this we calculated the number of days required for each species to germinate as well as the degree day sums above 0 °C in the soil. We counted the total number of shoots/leaves and maximum plant height within each experimental pot at the end of the first growing season. Following 'winter' we quantified any further germination and growth as described in the first growing season.

Timing and spatial scale

The experiment ran for two simulated Antarctic growing seasons with six months of simulated winter conditions (-5 °C in darkness) in between. The winter period is relevant as establishment of seedlings requires them to survive Antarctic winter conditions. Recording of germination at 3-4 day intervals was sufficient to capture first emergence and appropriate given the 6 month long growing seasons.

Data exclusions

no data were excluded

Reproducibility

The duration of this experiment (18 months) prevented us from conducting any realistic reproducibility procedures. However, there was a strong similarity in temperature requirements for germination between the two temperature treatments for all study species indicating that there was great consistency despite different provenance of seeds and plant families included.

Randomization	To avoid effects of placement within each chamber, pot positions were randomly repositioned every week and all pots were moved between chambers, every month to avoid any systematic 'chamber effect' throughout the experiment.
Blinding	Blinding was not possible as there were only two treatment chambers with clearly noticeable temperature differences for the person recording appearance of seedlings. Given that this is solely present/absence data there is no major issue regarding blinding procedures.

Did the study involve field work? [x] Yes [ ] No

Field work, collection and transport

Field conditions	Collection of organic material from the Antarctic
Location	Anchorage Island (Ryder Bay, south-east Adelaide Island, 67° 34' S 68° 07' W)
Access & import/export	The utmost care was taken to avoid disturbance of fragile Antarctic conditions by collecting minimal amounts of material from sites where there was clearly sufficient material present. Sampling permits were provided by Rijkswaterstaat (NL) and import to the UK was regulated through the British Antarctic Survey
Disturbance	The moss layer was lifted to collect organic material underneath but replaced when done. This will allow the alive moss layer to continue growing

Reporting for specific materials, systems and methods

We require information from authors about some types of materials, experimental systems and methods used in many studies. Here, indicate whether each material, system or method listed is relevant to your study. If you are not sure if a list item applies to your research, read the appropriate section before selecting a response.

Materials & experimental systems

n/a	Involved in the study
[x]	[ ] Antibodies
[x]	[ ] Eukaryotic cell lines
[x]	[ ] Palaeontology and archaeology
[ ]	[x] Animals and other organisms
[x]	[ ] Human research participants
[x]	[ ] Clinical data
[x]	[ ] Dual use research of concern

Methods

n/a	Involved in the study
[x]	[ ] ChIP-seq
[x]	[ ] Flow cytometry
[x]	[ ] MRI-based neuroimaging

Animals and other organisms

Policy information about studies involving animals; ARRIVE guidelines recommended for reporting animal research

Laboratory animals	The study did not involve animals
Wild animals	The study did not involve wild animals
Field-collected samples	We used soil obtained from beneath moss vegetation on Anchorage Island (Ryder Bay, south-east Adelaide Island, 67° 34' S 68° 07' W) collected during January 2018 and transported frozen (-20 °C) to laboratories in the Netherlands.<br>We used commercial walk-in cooling chambers (THEBO Horeca) with RIVA Cold refrigeration units (Rivacold srl – Vallefoglia, Italy). Growing season air temperatures were set to 2 °C and diurnal light intensity was modulated through light emitting diode (LED) lamps (Hortilight Sunfactor 270; 405 W) horizontally placed at 50 cm above pot height. The monthly mean diurnal light conditions recorded on Anchorage Island were used for 4 weeks of spring (2 weeks at October and November light conditions), 3 summer months (December, January, February light conditions) and 4 autumn weeks (2 weeks at March and April light conditions). During the last two weeks of the simulated growing season, the temperature in both chambers was lowered to 1 °C. After the simulated autumn, all plants were placed in a dark freezing chamber at -5 °C for six months, which simulates a typical Antarctic sub-nivean winter condition. Pots were sealed within plastic bags to limit freeze-desiccation during the winter period.
Ethics oversight	no ethical approval was required as vascular plants are not subject to such approval/regulations

Note that full information on the approval of the study protocol must also be provided in the manuscript.
